# Diseases of the Antrum or Maxilary Sinus

**Published:** 1859-04

**Authors:** F. D. Thurmond

**Affiliations:** Dentist, Atlanta, Ga.


### ARTICLE IV.

*Diseases of the Antrum or Maxilary Sinus.* By F. D. THURMOND, Dentist, Atlanta, Ga.

The existence of this cavity was but imperfectly known, previous to the middle of the seventeenth century, about which time a minute description of it was given.

Since which time anatomists and pathologists have had more than two hundred years to pry into its mysteries, and describe its diseases, and consequently, there are, in this our day, but few medical practitioners who are not well aware of the fact, that this cavity is liable to be the seat of many diseases; and though many of them are simple and manageable, some are malignant, dangerous, and often, fatal; bidding defiance to the most skillful treatment. Diseases of this concealed cavity, which are very simple in their nature, are often neglected, until the consequence arising from their peculiar situation, becomes ten-fold more alarming than the original disease, and often proves fatal. It is very important, therefore, that early attention be given to the first intimation of disease in this sinus.

I will now ask the indulgence of the readers, whilst I

attempt a short description of the various diseases of this cavity, commencing with the most simple:

*Retention of Mucus.*—The natural secretions of the mucus membrane of this cavity, pass off in the form of vapor, through the natural openings into the nasal cavity. But, when from any cause, which occasionally happens, these openings become closed, the secretion must accumulate in the cavity. As it accumulates it becomes more or less unhealthy and offensive. Finally, the cavity becomes entirely full; yet it does not cease to accumulate, but forces the bony walls to yield to its increasing bulk. The patient in many cases, is scarcely aware that anything is wrong, until the cavity is full.

But when the solid walls begin to yield, pain is the consequence, which becomes more severe as the accumulation increases. If this state of things is suffered to go on uninterrupted, the consequence is frequently alarming, especially with patients whose systems are tainted with other diseases. Though, as medical aid is almost invariably called in at this time, and immediate relief, if properly treated, at least from suffering, obtained.

We will give the treatment of this disease as it stands, and speak of the consequences under other heads, as the same consequences attend other diseases of this cavity. It is plain to be seen, that in the first place, the retained matter must be evacuated.

It has been recommended, and attempted by a few able surgeons, to pass a suitable instrument through the natural opening, into the antrum; but the success attending such efforts have been so unsatisfactory, that the practice has been, I believe, entirely abandoned. An opening should be be made into the antrum, and the most convenient and satisfactory plan, is to extract the second molar tooth, and perforate the sinus by passing an instrument through the alveolus of one of its roots. But if the second molar is sound and healthy, and the first or third one diseased, it would be better to select the diseased tooth than to sacrifice a healthy one. If the molar teeth are all sound, and one of the bicuspides is found to be decayed, the cavity may be entered from this point. though, not altogether as conveniently; it would, however, be advisable to put up with the inconvenience rather than to extract a sound tooth.

The proper tooth being selected, and extracted, a suitable instrument is to be passed into the alveolus of one of its roots, and gently and carefully, though firmly, passed thro' the bone into the cavity; care being taken not to let the instrument slip, and injure the opposite wall. After the cavity has been evacuated it is generally necessary to prevent the opening from closing by the use of a bougie or canula, confined, if necessary, to the adjacent teeth, until the interior of the cavity is rendered healthy by the use of suitable injections, &c., and the natural opening established. If the general health is bad, constitutional, remedies are to be resorted to; and if there are any diseased teeth which cannot be restored to health, they should all be extracted.

We will next make some remarks on Inflammation of the Lining Membrane of this Cavity.

[TO BE CONTINUED.]

